# Increased Expression of Lipid Metabolism Genes in Early Stages of Wooden Breast Links Myopathy of Broilers to Metabolic Syndrome in Humans

**DOI:** 10.3390/genes10100746

**Published:** 2019-09-25

**Authors:** Juniper A. Lake, Michael B. Papah, Behnam Abasht

**Affiliations:** 1 Center for Bioinformatics and Computational Biology, University of Delaware, Newark, DE 19711, USA; dnovick@udel.edu; 2 Department of Animal and Food Sciences, University of Delaware, Newark, DE 19716, USA; papah@udel.edu

**Keywords:** wooden breast, broilers, myopathy, breast muscle, meat quality

## Abstract

Wooden breast is a muscle disorder affecting modern commercial broiler chickens that causes a palpably firm pectoralis major muscle and severe reduction in meat quality. Most studies have focused on advanced stages of wooden breast apparent at market age, resulting in limited insights into the etiology and early pathogenesis of the myopathy. Therefore, the objective of this study was to identify early molecular signals in the wooden breast transcriptional cascade by performing gene expression analysis on the pectoralis major muscle of two-week-old birds that may later exhibit the wooden breast phenotype by market age at 7 weeks. Biopsy samples of the left pectoralis major muscle were collected from 101 birds at 14 days of age. Birds were subsequently raised to 7 weeks of age to allow sample selection based on the wooden breast phenotype at market age. RNA-sequencing was performed on 5 unaffected and 8 affected female chicken samples, selected based on wooden breast scores (0 to 4) assigned at necropsy where affected birds had scores of 2 or 3 (mildly or moderately affected) while unaffected birds had scores of 0 (no apparent gross lesions). Differential expression analysis identified 60 genes found to be significant at an FDR-adjusted *p*-value of 0.05. Of these, 26 were previously demonstrated to exhibit altered expression or genetic polymorphisms related to glucose tolerance or diabetes mellitus in mammals. Additionally, 9 genes have functions directly related to lipid metabolism and 11 genes are associated with adiposity traits such as intramuscular fat and body mass index. This study suggests that wooden breast disease is first and foremost a metabolic disorder characterized primarily by ectopic lipid accumulation in the pectoralis major.

## 1. Introduction

Wooden breast is one of several muscle abnormalities of modern commercial broiler chickens that causes substantial economic losses in the poultry industry due to its impact on meat quality. Emerging evidence suggests wooden breast may also be detrimental to bird welfare as affected chickens exhibit increased locomotor difficulties, decreased wing mobility, and higher mortality rates [1,2,3]. While the etiology of the myopathy is still poorly understood, many believe it to be a side-effect of improved management practices and selective breeding for performance traits due to increased susceptibility among broilers with high feed efficiency [4,5], breast muscle yield [4,6,7], and growth rate [8,9]. 

Macroscopic manifestations of the disorder include pale and hardened areas, subcutaneous and fascial edema, petechial hemorrhages, spongy areas with disintegrating myofiber bundles, and white fatty striations characteristic of white striping [2,10]. An early study of wooden breast characterized its microscopic presentation as polyphasic myodegeneration and necrosis with regeneration and interstitial connective tissue accumulation (fibrosis), primarily affecting the cranial end of the pectoralis major muscle [10]. However, it has since been demonstrated that venous inflammation (phlebitis) and perivascular lipid and inflammatory cell infiltration appear in the first week of age and precede other symptoms [2]. Differential gene expression analysis of the pectoralis major in 7-week-old broilers suggests that hypoxia, oxidative stress, fiber-type switching, and increased intracellular calcium may be important components of the myopathy [11]. In two- and three-week old birds, differentially expressed genes were mostly associated with increased inflammation, vascular disease, increased oxidative stress, extracellular matrix remodeling, dysregulation of carbohydrates and lipids, and impaired excitation-contraction coupling [12]. Metabolomic profiling is in agreement with these results and provides evidence of oxidative stress and dysregulated carbohydrate and lipid metabolism in affected birds at 7 weeks of age [13].

The objective of the present study was to better characterize the transcriptional anomalies that exist in the pectoralis major of two-week-old birds that later develop wooden breast by market age at 7 weeks. Only one other gene expression study has investigated early stages of wooden breast [12]. The current study serves as a continuation of that work, but makes two key changes. First, unlike the previous study that used only male birds, we included only female birds in the RNA-seq analysis. Second, birds in the affected group all possessed mild or moderate wooden breast phenotypes rather than severe symptoms, which allowed us to capture a clearer signal of the earliest transcriptomic perturbations associated with the myopathy.

## 2. Materials and Methods 

### 2.1. Experimental Animals and Tissue Collection

The University of Delaware Institutional Animal Care and Use Committee approved the animal conditions and experimental procedures used in this scientific study under protocol number 48R-2015-0. For this experiment, 302 mixed male and female Cobb500 broilers were raised according to industry growing standards in two poultry houses from 1 day to 7 weeks of age. Birds were divided between two poultry houses due to capacity constraints, but both houses were maintained at the same environmental conditions. Chickens were provided with continuous free access to water and feed that met all nutritional recommendations for Cobb500 broilers. At 14 days of age, biopsy samples of the craniolateral area of the left pectoralis major muscle were collected in the same manner described by a previous study [12] from 101 birds randomly selected from both houses. After biopsy, all birds were grown out to 7 weeks of age, at which time they were euthanized by cervical dislocation. During necropsy, the pectoralis major muscles were evaluated for gross lesions and palpable firmness associated with wooden breast and each bird was assigned a wooden breast score using a 0-4 scale; 0-Normal indicates the bird had no macroscopic signs of the myopathy, 1-Very Mild indicates 1% or less of the breast muscle was affected, 2-Mild indicates between 1% and 10% of the breast muscle was affected, 3-Moderate indicates between 10% and 50% of the breast muscle was affected, and a score of 4-Severe indicates that more than 50% was affected. This scoring system is slightly different from the one previously used in our laboratory and separates unaffected and mildly and moderately affected chickens with higher resolution.

### 2.2. Sample Selection and RNA-Sequencing 

Selection of samples for use in RNA-seq analysis was based on wooden breast scores assigned at necropsy at 7 weeks of age. A total of 6 unaffected and 8 affected birds were identified; affected birds had scores of 2 or 3 (mildly or moderately affected) while unaffected birds had scores of 0 (no apparent gross lesions). Only samples taken from female birds were used for RNA-seq. Total RNA was extracted from pectoralis major tissue samples using the mirVana miRNA Isolation Kit (Thermo Fisher Scientific, Waltham, MA, USA) according to the manufacturer’s protocol and stored at −80 °C until cDNA library preparation. Each RNA sample was quantified using the NanoDrop 1000 Spectrophotometer (Thermo Fisher Scientific) and quality was assessed with the Fragment Analyzer at the Delaware Biotechnology Institute (DBI). cDNA libraries were constructed using the ScriptSeq Complete Kit (Human/Mouse/Rat) (Illumina, San Diego, CA, USA) with the optional step of adding a user-defined barcode to the library. The 14 barcoded cDNA libraries were normalized and 10 µl of each sample were pooled in two tubes (7 samples in each pool). Pooled libraries were subsequently submitted to the DBI for paired-end 2 × 76-nucleotide sequencing on two lanes of a flow cell using the HiSeq 2500 Sequencing System (Illumina). 

Raw sequencing reads were demultiplexed and then checked for quality using FastQC v0.11.7 [14]. All samples passed the quality check and were submitted to Trimmomatic v0.38 [15] to trim leading and trailing bases with quality below 20, remove reads with an average quality below 15, and remove reads that were shorter than 30 bases in length. Trimmed reads were then mapped to both Gallus_gallus-5.0 (Ensembl release 94) and GRCg6a (Ensembl release 95) chicken reference genomes using HISAT2 v2.1.0 [16] with concordant mapping required for both reads in each pair. Cuffdiff v2.2.1 [17] was used with the fragment bias correction option to identify differentially expressed genes between affected and unaffected birds. Genes were considered statistically significant if the FDR-adjusted *p*-value was ≤ 0.05. The use of two reference genome builds, the latter of which was released during the course of this study, provided validation of our results and allowed us to capture differentially expressed genes that may have borderline statistical significance due to assembly errors or bias. One sample (animal ID 424183) in the unaffected group displayed an extreme outlier expression pattern; it was therefore removed and differential expression analysis with Cuffdiff was repeated without this sample (5 unaffected vs. 8 affected). To compile the results generated from each reference genome, Ensembl Gene IDs from Gallus-gallus-5.0 were mapped to GRCg6a gene IDs using Ensembl’s ID History Converter; differentially expressed genes with annotation differences between the two reference genome releases were scrutinized for consistency. Pairwise correlation analysis and visualization of differentially expressed genes was conducted with the “stats” and “corrplot” packages [18] in R only using expression data generated with the GRCg6a reference genome build. 

## 3. Results

An average of 19,616,353 paired-end sequence reads were generated per sample, which was reduced to an average of 19,609,044 paired-end reads after trimming. The average mapping rate per sample was 74.5% with the Gallus_gallus-5.0 reference genome build and 75.1% with GRCg6a. The total number of sequenced reads, trimmed reads, and mapped reads per sample can be found in Table S1.

There were 52 differentially expressed genes identified using the Gallus_gallus-5.0 reference genome build and 29 differentially expressed genes using the GRCg6a genome build. After accounting for changes in annotation of Ensembl Gene IDs between genome releases, a total of 60 genes were found to be differentially expressed between affected and unaffected groups across both analyses, with 18 differentially expressed genes overlapping between both Gallus_gallus-5.0 and GRCg6a. Three Ensembl Gene IDs from the earlier build were deprecated in GRCg6a and were excluded from further analysis. Of the 60 differentially expressed genes used for downstream analysis, 52 were up-regulated in affected birds and 8 were down-regulated in affected birds (Table 1).

**Figure 1:**
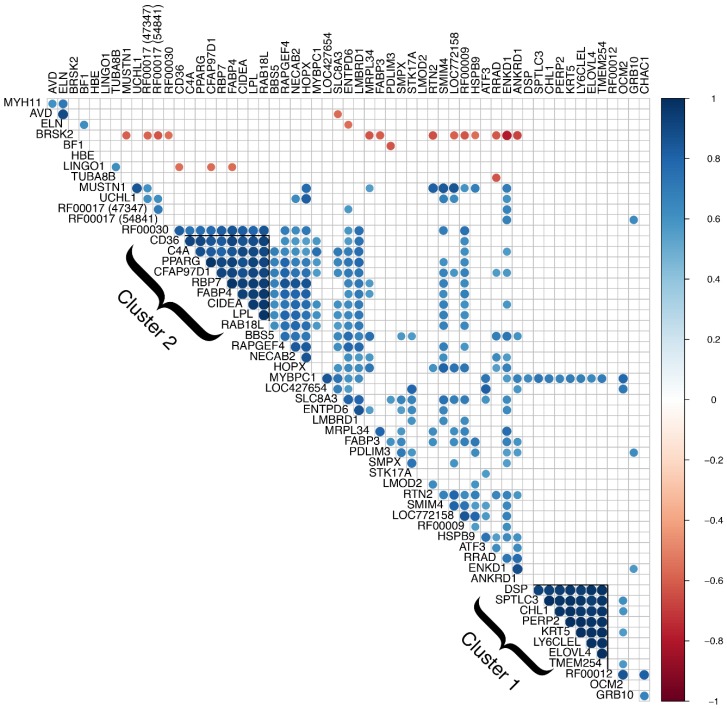
Correlation analysis of differentially expressed genes. Genes with significantly correlated expression (*p-*value ≤ 0.05) are shown in blue (positive correlation) and red (negative correlation). Two major clusters of genes have Pearson’s correlation coefficients greater than 0.8 for all gene pairs. Cluster 1 consists of 8 genes, all of which were excluded from further analysis due to presumed skin contamination. Cluster 2 consists of 9 genes related to lipid metabolism or adiposity traits.

Correlation analysis of differentially expressed genes revealed two major clusters with Pearson’s correlation coefficients greater than 0.8 for all gene pairs (see Figure 1). The first is a cluster of 8 genes, all of which were excluded from further analysis due to presumed skin contamination. These include *serine palmitoyltransferase long-chain base subunit 3* (*SPTLC3*), *desmoplakin* (*DSP*), *ELOVL fatty acid elongase 4* (*ELOVL4*), *PERP2, TP53 apoptosis effector* (*PERP2*), *keratin 5* (*KRT5*), *cell adhesion molecule L1* like (*CHL1*), *lymphocyte antigen 6 complex, locus E-like* (*LY6CLEL*), and *transmembrane protein 254* (*TMEM254*). Several of these genes are known to be primarily expressed in the skin and a previous biopsy study using the same technique demonstrated that biopsy samples are prone to skin contamination [12]. Additionally, differential expression of these genes was driven by the same three samples, one unaffected and two affected, and expression in the remaining samples was relatively very low or approximately zero. The second cluster consisted of 9 protein-coding genes with demonstrated or putative involvement in lipid metabolism. Although no other clusters were apparent from correlation analysis, functional groupings of differentially expressed genes included muscle growth and function, calcium signaling, and endoplasmic reticulum (ER) stress response. We also found a substantial number of genes that are differentially expressed, implicated, or otherwise involved in metabolic syndrome in mammals, which is characterized primarily by diabetes, insulin resistance, obesity, elevated blood lipids, and high blood pressure.

## 4. Discussion

Metabolic syndrome refers to a cluster of conditions, including obesity, high blood sugar, high serum triglycerides, low serum HDL (high-density lipoprotein) cholesterol, and high blood pressure, that put an individual at greater risk of developing type 2 diabetes and associated complications such as atherosclerosis, cardiomyopathy, non-alcoholic fatty liver disease, and diabetic nephropathy. Our data revealed a surprising number of differentially expressed genes implicated in or associated with metabolic syndrome in humans. Among the 60 differentially expressed genes identified in this study, 20 are previously reported to exhibit altered expression in relation to diabetes or a closely related metabolic condition and nine genes have been identified as candidate genes in association studies of glucose tolerance or diabetes mellitus (Table 2). One of these candidate genes, *Bardet-Biedl syndrome 5* (*BBS5*), is associated with a rare ciliopathy that strongly predisposes individuals to diabetes and other metabolic complications: obesity and diabetes mellitus are actually considered diagnostic features of the disease [19,20]. Upon further examination, we found that many of the conditions surrounding metabolic syndrome in humans possessed important similarities to the wooden breast phenotype, namely inflammation, ectopic fat deposition, dysregulation of Ca^2+^ homeostasis, ER stress, oxidative stress, altered glucose metabolism, fibrosis, and hypertrophy. 

### 4.1. Increased Expression of Genes Involved in Lipid Metabolism

The most important connection to metabolic syndrome in our results is the increased expression of genes involved in lipid metabolism in the pectoralis major of affected birds. Many of the differentially expressed genes from our analysis encode proteins with critical or rate-limiting functions in lipid metabolism and homeostasis such as lipoprotein triglyceride hydrolysis, fatty acid transport, and lipid droplet regulation. These genes include *lipoprotein lipase* (*LPL*), *CD36 molecule* (*CD36*), *peroxisome proliferator-activated receptor gamma* (*PPARG*), *retinol-binding protein 7* (*RBP7*), *fatty acid-binding protein 3* (*FABP3*), fatty *acid-binding protein 4* (*FABP4*), *cell death-inducing DFFA-like effector a* (*CIDEA*), *Ras-related protein Rab-18-B-like* (*RAB18L*), and *LMBR1 domain containing 1* (*LMBRD1*) [31,48,52,53,54,55,56,57,58,59,60]. An outstanding member of this group is *LPL*, which encodes lipoprotein lipase, a rate-limiting catalyst for hydrolysis of the triglycerides found in circulating lipoproteins, providing free fatty acids and monoglycerides for use by surrounding tissues [52,53]. LPL serves as a metabolic gatekeeper in terms of partitioning circulating lipids among tissues, with highest activity in tissues that oxidize or store large quantities of fatty acids [53]. Regulation of *LPL* expression occurs in a tissue-specific manner and alterations to LPL activity in one tissue can affect systemic nutrient partitioning by reducing substrate (lipid) availability to other tissues [53].

Several other genes, some of which are functionally uncharacterized or poorly understood regarding lipid metabolism, have expression or genetic polymorphisms correlated with adiposity traits such as body mass index, percent intramuscular fat, percent abdominal fat, or blood lipid levels. These include *HOP homeobox* (*HOPX*), *myosin-binding protein-C, slow type* (*MYBPC1*), *Bardet-Biedl syndrome 5* (*BBS5*), *growth factor receptor bound protein 10* (*GRB10*), *CFAP97 domain containing 1* (*CFAP97D1*), *hemoglobin subunit epsilon* (*HBE*), *ectonucleoside triphosphate diphosphohydrolase 6* (ENTPD6), *complement C4A (Rodgers blood group)* (*C4A*), *mitochondrial ribosomal protein L34* (*MRPL34*), *ATF3*, and *CHAC1* [20,61,62,63,64,65,66,67,68,69,70,71,72,73,74].

Notably, the present study identified a cluster of nine genes related to lipid metabolism that may represent a functional group for two main reasons. First, the genes in this cluster exhibit highly correlated expression (*r* > 0.8 for all gene pairs; see Figure 1). Second, this cluster includes the gene encoding the transcription factor PPARγ and several of its experimentally validated transcriptional targets (*CD36*, *C4A*, *RBP7*, *FABP4*, *CIDEA*, and *LPL*) [55,56,61,75,76]. As a master regulator of adipogenesis, PPARγ plays a crucial role in governing the distribution of lipid deposition in the body and the development of various metabolic conditions [77,78,79,80,81]. It is also one of the few established genes that has been associated with common forms of type 2 diabetes across multiple genome-wide association studies [43,44,45,46,47]. In skeletal muscle of broiler chickens and other meat-type animals, increased expression of *PPARG* and PPARγ target genes is frequently associated with higher intramuscular fat content [82,83,84,85]. 

Increased expression of genes related to lipid metabolism and fat deposition in the pectoralis major may signify the pathological overprovisioning of lipids to the muscle, consistent with histological characterization of early stages of wooden breast, in which lipid infiltration and accumulation was established as one of the first signs of disease even before wooden breast is grossly detectable [2]. The storage of excess lipids in tissues other than adipose tissue, that normally contain only small amounts of fat, is called ectopic lipid deposition and is linked to insulin resistance and metabolic dysfunction in mammals [86]. In fact, ectopic lipid deposition and the resulting lipotoxicity are considered to be a major feature of metabolic syndrome with the precise location of ectopic lipid accumulation dictating specific complications such as atherosclerosis, hepatic steatosis, and diabetic nephropathy [87]. This suggests that increased lipid deposition in the pectoralis major may be a major factor contributing to the wooden breast phenotype.

### 4.2. Endoplasmic Reticulum Stress and Dysregulation of Calcium Homeostasis

Results from the current study suggest that ER stress and dysregulation of calcium homeostasis are occurring in the pectoralis major muscle in the early stages of wooden breast. Evidence for ER stress is supported by the up-regulation of *activating transcription factor 3* (*ATF3*), *ChaC glutathione specific gamma-glutamylcyclotransferase 1* (*CHAC1*), and *reticulon 2* (*RTN2*) and the down-regulation of *BR serine/threonine kinase 2* (*BRSK2*). *ATF3*, *CHAC1,* and *BRSK2* are part of the unfolded protein response [88,89], a highly conserved cellular stress response caused by an accumulation of unfolded or misfolded proteins in the ER [90]. An association between the unfolded protein response, lipid metabolism, dysregulation of calcium homeostasis, and metabolic syndrome has been clearly established, but the direction of causality is controversial [91,92,93,94]. The role of ATF3 in particular has been studied in the context of type 2 diabetes, non-alcoholic fatty liver disease, diabetic cardiomyopathy, atherosclerosis, and obesity, with some authors suggesting it may have both detrimental and beneficial functions related to insulin resistance, mitochondrial dysfunction, and inflammation in response to high-fat diets [22,67,95,96,97,98,99,100]. In arterial endothelial cells, ATF3 expression can be induced by exposure to high levels of triglyceride-rich lipoprotein lipolysis products [96], substantiating it as a link between high lipid metabolism and cellular stress response.

One of the most compelling links to metabolic syndrome and dysregulation of calcium homeostasis in our data is *Ras-related glycolysis inhibitor and calcium channel regulator* (*RRAD*), a gene encoding a small GTPase that binds directly to Ca^2+^ channel beta subunits to regulate intracellular Ca^2+^ signaling in muscle cells [101]. RRAD also has regulatory functions via its interaction with calmodulin [102]. This gene was originally named *Ras-related associated with diabetes* because it was identified via subtraction cloning as the only gene out of 4000 cDNA clones that was overexpressed in skeletal muscle of type 2 diabetic individuals compared to non-diabetic or type 1 diabetic individuals [26]. Overexpression of *RRAD* in cultured myocytes was found to reduce insulin-stimulated glucose uptake by 50–90%, which the authors speculated was due to a decrease in intrinsic activity of glucose transporter 4, the insulin-dependent glucose transporter [103]. An in vivo study of transgenic mice overexpressing *RRAD* in skeletal muscle found that high-fat feeding produced not only insulin resistance in transgenic mice, but also increased triglyceride metabolism compared to controls [104]. This suggests that RRAD may inhibit glycolysis independently from its action on glucose transporters via substrate competition with fatty acids [105]. Another regulator of intracellular Ca^2+^, *RAPGEF4*, up-regulated in the current study, is the direct target of some anti-diabetic drugs called sulfonylureas [49]. 

The role of Ca^2+^ in metabolic syndrome and diabetes is complex and not fully understood, but one theory suggests that the disruption of Ca^2+^ homeostasis is a feed-forward pathological cycle resulting from ER dysfunction during chronic exposure to excessive nutrients and energy [106]. The majority of intracellular Ca^2+^ pools are contained within the ER, known as the sarcoplasmic reticulum in muscle cells; however, high concentrations of fatty acids can mediate a substantial redistribution of ER luminal Ca^2+^ stores among subcompartments of the ER and from the ER to the cytosol, leading to ER stress and eventually cell death [107,108].

### 4.3. Increased Expression of Genes Related to Hypertrophy and Slow-Twitch Muscle

Several genes involved in myogenic differentiation and muscle hypertrophy are up-regulated in affected birds: *musculoskeletal, embryonic nuclear protein 1* (*MUSTN1*), *ankyrin repeat domain 1* (*ANKRD1*), and *HOPX* have roles in myotube formation, myofusion, and regulation of other myoblast differentiation genes [72,109,110,111,112,113], although studies of *HOPX* in chickens have found that it is most highly expressed in adipose tissue and has functions related to adipocyte differentiation [71,72]. Other up-regulated genes related to development and regeneration of the musculosketal system include *PDZ and LIM domain 3* (*PDLIM3*), *small muscle protein X-linked* (*SMPX*), and *leiomodin 2* (*LMOD2*). Two of these, *SMPX* and *PDLIM3,* encode Z-disc associated proteins with putative mechanosensory or stretch signaling roles in striated muscle [114,115]. Expression of *MUSTN1*, *ANKRD1*, *PDLIM3,* and *SMPX* can be induced by eccentric contraction exercises [116,117,118] or passive stretch [119], suggesting a role in muscle hypertrophy and repair [115]. Although some of these genes possess roles in muscle fiber regeneration, we do not believe that they are indicative of the regenerative process that characterizes later stages of wooden breast. Regeneration of new muscle fibers occurs in response to degeneration and necrosis and is not apparent microscopically until 3 weeks of age [2].

Up-regulation of genes related to hypertrophy in affected birds is in line with higher breast muscle yield in affected birds [4,6,7]. In fact, speculation on the cause of wooden breast and related muscle disorders has focused largely on impaired oxygen supply and buildup of metabolic waste resulting from sustained rapid growth of the pectoralis major [120,121,122,123]. However, up-regulation of genes involved in hypertrophy may also be part of the disease process, causing excessive growth of pectoralis major in affected chickens. Considering the metabolic and physiologic similarities between wooden breast and diabetes, it is possible that mechanisms underlying muscle hypertrophy in wooden breast are similar to those that cause hypertrophy of organs in diabetic complications. For example, diabetic cardiomyopathy, non-alcoholic fatty liver disease, and diabetic nephropathy can cause structural remodeling that includes hypertrophy of the heart, liver, and kidneys respectively [124,125,126].

Interestingly, our data showed up-regulation of several genes, such as *MYBPC1* and *SMPX* [127,128], that are more closely associated with slow-twitch oxidative muscle rather than fast-twitch glycolytic muscle. For example, *LMOD2* has been alternatively called *cardiac leiomodin* and its levels in cardiac muscle are directly linked to the length of actin-containing thin filaments due to competition for binding with tropomodulin-1 [129]. Overexpression of *LMOD2* in the heart results in elongation of thin filaments and reduced cardiac function as proper thin filament length is necessary to generate contractile force [129,130]. Similarly, *ANKRD1* was previously named *cardiac ankyrin repeat protein* (*CARP*) and has been proposed as a marker of cardiac hypertrophy due to its increased expression in three distinct models of cardiac hypertrophy in rats [131]. The lipid transporter *FABP3*, which is involved in the uptake, intracellular metabolism, and transport of long-chain fatty acids, is most abundantly expressed in slow-twitch skeletal and cardiac muscle of humans rather than fast-twitch skeletal muscle [66]. Up-regulation of these genes is consistent with previous reports of fiber-type switching in 7-week-old birds with wooden breast [11] and may suggest that the pectoralis major muscle of affected birds resembles cardiac or slow-twitch muscle at the transcriptional level.

### 4.4. Comparison with Prior Gene Expression Study of Early Stages of Wooden Breast

A considerable number of differentially expressed genes from the current study were previously identified by Papah et al. [12], who studied the early pathogenesis of wooden breast in male broilers. Of the 20 genes that were previously identified, the majority were found to be differentially expressed in 3-week-old birds rather than 2-week-old birds (Table 3). A key difference between these studies is the use of male birds in the previous study and female birds in the present experiment. Methodological discrepancies, such as broiler line and severity of disease in affected birds, prevent us from drawing conclusions from these two studies about sex-linked differences in global gene expression that might help explain the higher prevalence and severity of wooden breast among male birds compared to females [8]. However, it has been suggested that increased expression of genes related to fat metabolism and deposition in the pectoralis major of male broilers at 3 weeks of age may contribute to their increased susceptibility [132]. Incomplete dosage compensation in male broilers, which are homogametic and possess two Z chromosomes, may also play an important role [132]. The only Z-linked gene found to be differentially expressed in our results is *LPL*, which encodes a rate-limiting catalyst for lipoprotein triglyceride hydrolysis [52,53]. Up-regulation of *LPL* in the pectoralis major of affected birds may increase the rate that lipids are taken up by the muscle, making it a critical gene for ectopic lipid deposition.

Our results highlight the importance of increased lipid metabolism in governing susceptibility to wooden breast and establish high expression of lipid metabolism genes as a much earlier signature of the disease than was previously believed [12]. This is consistent with previous reports of metabolic perturbations in wooden breast [11,12,13], especially a recent study suggesting that the increased ability to direct alimentary resources, particularly fatty acids, to the pectoralis major muscle may underlie susceptibility to wooden breast [5]. While our findings reveal strong parallels between wooden breast in broilers and metabolic syndrome in humans, it is important to recognize that wooden breast is not associated with increased visceral adiposity [5,133] or elevated blood glucose levels [9]. Additionally, lipotoxicity in human skeletal muscle causes muscle atrophy [134] while wooden breast-affected birds have a significantly larger pectoralis major muscle compared to unaffected birds [6]. It is important to investigate how ectopic lipid accumulation in skeletal muscle can manifest with such disparate phenotypes in humans compared to broilers.

## 5. Conclusions

The findings of this study show that transcriptional changes associated with early stages of wooden breast disease in 2-week-old birds have significant overlap with genes that are dysregulated in metabolic syndrome in humans. Although the underlying causes of metabolic dysfunction possibly leading to pathological progression of wooden breast remain unknown, this study clearly demonstrates that early up-regulation of lipid metabolism in the pectoralis major is a key feature of the myopathy. Additionally, the finding that PPARγ and several of its transcriptional target genes are expressed higher in affected chickens provides critical insight into the early pathogenesis of wooden breast. Affected birds also show dysregulation of various genes involved in muscle growth and function as well as calcium signaling and ER stress. Additional research is needed to understand the mechanisms underlying the apparent metabolic dysfunction and to investigate the possible link between wooden breast and metabolic syndrome. 

## Figures and Tables

**Table 1:** Differentially expressed genes between wooden breast-affected pectoralis major muscle samples and unaffected samples at 2 weeks of age. Log2FC is calculated by log2(FPKMaffected/FPKMunaffected). Unknown gene names are indicated with a dash (-). Non-significant *p*-values (i.e., FDR-adjusted *p*-values > 0.05) are indicated as n.s.

Gene ID	Gene Symbol	Gene Name	Log2FC Galgal5	Log2FC GRCg6a
**Genes up-regulated in affected group**				
ENSGALG00000046652	-	-	1.55	n.s.
ENSGALG00000052084	-	-	n.s.	2.06
ENSGALG00000006491	ANKRD1	Ankyrin repeat domain 1	1.28	0.99
ENSGALG00000049422 *	ATF3	Activating transcription factor 3	1.04	n.s.
ENSGALG00000009846	BBS5	Bardet-Biedl syndrome 5	0.76	n.s.
ENSGALG00000017040	C4A	Complement C4A (Rodgers blood group)	1.24	0.96
ENSGALG00000008439	CD36	CD36 molecule	0.87	n.s.
ENSGALG00000046316	CFAP97D1	CFAP97 domain containing 1	1.14	n.s.
ENSGALG00000027874	CHAC1	ChaC glutathione specific gamma-glutamylcyclotransferase 1	1.99	n.s.
ENSGALG00000037856	CHL1	Cell adhesion molecule L1 like	1.44	n.s.
ENSGALG00000034500	CIDEA	Cell death-inducing DFFA-like effector a	1.51	1.22
ENSGALG00000012790	DSP	Desmoplakin	1.50	n.s.
ENSGALG00000015876	ELOVL4	ELOVL fatty acid elongase 4	2.19	n.s.
ENSGALG00000001204	ENKD1	Enkurin domain containing 1	2.07	n.s.
ENSGALG00000008563	ENTPD6	Ectonucleoside triphosphate diphosphohydrolase 6	0.80	n.s.
ENSGALG00000037050	FABP3	Fatty acid-binding protein 3	0.77	0.72
ENSGALG00000030025	FABP4	Fatty acid-binding protein 4	1.74	1.52
ENSGALG00000013100	GRB10	Growth factor receptor bound protein 10	0.77	n.s.
ENSGALG00000011404	HOPX	HOP (homeodomain-only protein) homeobox	1.19	1.04
ENSGALG00000023818	HSPB9	Heat shock protein family B (small) member 9	1.14	1.13
ENSGALG00000032672	KRT5	Keratin 5	n.s.	1.68
ENSGALG00000016174	LMBRD1	LMBR1 domain containing 1	0.87	n.s.
ENSGALG00000008805	LMOD2	Leiomodin 2	1.39	n.s.
ENSGALG00000021286	LOC427654	Parvalbumin beta-like	2.36	2.36
ENSGALG00000023819	LOC772158	Heat shock protein 30C-like	0.74	0.76
ENSGALG00000015425	LPL	Lipoprotein lipase	0.80	n.s.
ENSGALG00000043582	LY6CLEL	Lymphocyte antigen 6 complex, locus E-like	2.25	n.s.
ENSGALG00000036004	MRPL34	Mitochondrial ribosomal protein L34	n.s.	0.82
ENSGALG00000001709	MUSTN1	Musculoskeletal, embryonic nuclear protein 1	n.s.	1.30
ENSGALG00000012783	MYBPC1	Myosin-binding protein-C, slow type	1.49	1.17
ENSGALG00000003323	NECAB2	N-terminal EF-hand calcium binding protein 2	n.s.	1.10
ENSGALG00000053246 *	OCM2	Oncomodulin 2	0.78	n.s.
ENSGALG00000013414	PDLIM3	PDZ and LIM domain 3	0.75	n.s.
ENSGALG00000027207	PERP2	PERP2, TP53 apoptosis effector	1.29	n.s.
ENSGALG00000004974	PPARG	Peroxisome proliferator-activated receptor gamma	0.89	n.s.
ENSGALG00000040434	RAB18L	Ras-related protein Rab-18-B-like	1.23	1.09
ENSGALG00000043694	RAPGEF4	Rap guanine nucleotide exchange factor 4	1.24	n.s.
ENSGALG00000002637	RBP7	Retinol-binding protein 7	1.96	1.65
ENSGALG00000025650	RF00009	Ribonuclease P RNA component H1, 2 pseudogene	n.s.	0.75
ENSGALG00000051839	RF00012	-	n.s.	2.02
ENSGALG00000047347 *	RF00017	-	1.38	n.s.
ENSGALG00000025557	RF00030	-	n.s.	0.88
ENSGALG00000054841*	RF0017	-	0.83	1.46
ENSGALG00000005140	RRAD	RRAD (Ras-related associated with diabetes), Ras-related glycolysis inhibitor and calcium channel regulator	1.63	1.20
ENSGALG00000051456	RTN2	Reticulon 2	n.s.	1.00
ENSGALG00000009400	SLC8A3	Solute carrier family 8 member A3	0.70	n.s.
ENSGALG00000042863	SMIM4	Small integral membrane protein 4	n.s.	0.87
ENSGALG00000019157	SMPX	Small muscle protein X-linked	0.94	n.s.
ENSGALG00000009037	SPTLC3	Serine palmitoyltransferase long-chain base subunit 3	2.47	n.s.
ENSGALG00000031117	STK17A	Serine/threonine kinase 17a	0.92	n.s.
ENSGALG00000021231	TMEM254	Transmembrane protein 254	1.05	n.s.
ENSGALG00000014261	UCHL1	Ubiquitin C-terminal hydrolase L1	1.07	0.94
**Genes down-regulated in affected group**				
ENSGALG00000025945 *	AVD	Avidin	−1.58	−1.69
ENSGALG00000033932	BF1	MHC BF1 class I	−0.84	n.s.
ENSGALG00000006681	BRSK2	BR serine/threonine kinase 2	−2.50	−2.77
ENSGALG00000032220	ELN	Elastin	n.s.	−1.17
ENSGALG00000035309	HBE	Hemoglobin subunit epsilon	−1.59	n.s.
ENSGALG00000002708	LINGO1	Leucine-rich repeat and immunoglobulin-like domain containing nogo receptor-interacting protein 1	−0.78	−0.77
ENSGALG00000006520	MYH11	Myosin, heavy chain 11, smooth muscle	−0.95	n.s.
ENSGALG00000013045	TUBA8B	Tubulin, alpha 8b	−1.67	n.s.

* These genes had annotation differences between Gallus_gallus-5.0 and GRCg6a reference genome assemblies.

**Table 2:** Differentially expressed genes linked to diabetes and glucose tolerance. Of the 60 differentially expressed genes identified in this study, 26 are either proposed as candidate genes for glucose tolerance or diabetes mellitus or exhibit altered expression in relation to diabetes or a closely related metabolic condition.

Gene Symbol	Gene Name	Connection	Sources
ANKRD1	Ankyrin repeat domain 1	Expression	[21]
ATF3	Activating transcription factor 3	Expression	[22]
BBS5	Bardet-Biedl syndrome 5	Genetic variant	[19]
BF1	MHC BF1 class I	Expression	[23]
BRSK2	BR serine/threonine kinase 2	Expression	[24]
C4A	Complement C4A (Rodgers blood group)	Expression	[25]
C4A	Complement C4A (Rodgers blood group)	Genetic variant	[24]
CD36	CD36 molecule	Expression	[26,27]
CIDEA	Cell death-inducing DFFA-like effector a	Expression	[28]
ENTPD6	Ectonucleoside triphosphate diphosphohydrolase 6 (putative)	Genetic variant	[29]
FABP3	Fatty acid-binding protein 3	Expression	[30]
FABP4	Fatty acid-binding protein 4	Expression	[30,31,32]
GRB10	Growth factor receptor bound protein 10	Expression	[33,34]
LINGO1	Leucine-rich repeat and immunoglobulin-like domain containing nogo receptor-interacting protein 1	Genetic variant	[35,36]
LMBRD1	LMBR1 domain containing 1	Expression	[27]
LMOD2	Leiomodin 2	Genetic variant	[37]
LPL	Lipoprotein lipase	Expression	[32,38]
LPL	Lipoprotein lipase	Genetic variant	[39]
MRPL34	Mitochondrial ribosomal protein L34	Expression	[40,41]
MYH11	Myosin, heavy chain 11, smooth muscle	Expression	[26]
PDLIM3	PDZ and LIM domain 3	Genetic variant	[37]
PPARG	Peroxisome proliferator-activated receptor gamma	Expression	[32,42]
PPARG	Peroxisome proliferator-activated receptor gamma	Genetic variant	[43,44,45,46,47]
RAB18L	Ras-related protein Rab-18-B-like	Expression	[48]
RAPGEF4	Rap guanine nucleotide exchange factor 4	Expression	[49]
RBP7	Retinol-binding protein 7	Expression	[50]
RRAD	RRAD, Ras-related glycolysis inhibitor and calcium channel regulator	Expression	[26]
RTN2	Reticulon 2	Genetic variant	[24]
UCHL1	Ubiquitin C-terminal hydrolase L1	Expression	[51]

**Table 3:** Comparison of differentially expressed genes with previous study of wooden breast in male broilers. A total of 20 genes from the current study were also previously identified at early stages of wooden breast development in 2- and 3-week-old male broilers by Papah et al. [12].

Biopsy Age	No. Genes	Gene Symbols
2 weeks	4	*ANKRD1, ATF3, CHAC1, RAPGEF4*
3 weeks	18	*AVD, BBS5, CD36, CHAC1, CIDEA, FABP4, HOPX, LINGO1, LMOD2, LPL, MYBPC1, OCM2, RAPGEF4, RBP7, RRAD, SMPX, STK17A, UCHL1*

## Supplementary Materials

The following are available online at https://www.mdpi.com/2073-4425/10/10/746/s1, Table S1: Sequencing and mapping statistics for RNA-Seq analysis of eight wooden breast-affected and six unaffected pectoralis major muscle samples.
