# Supplementary material for: Increased Expression of Lipid Metabolism Genes in Early Stages of Wooden Breast Links Myopathy of Broilers to Metabolic Syndrome in Humans
Source: Genes (Basel). 2019 Sep 25;10(10):746. doi: 10.3390/genes10100746 (PMC6826700; doi:10.3390/genes10100746)
Supplement: Supplementary file 1 [file genes-10-00746-s001.pdf]

Table S1. Sequencing and mapping statistics for RNA-Sequencing of 8 wooden breast affected and 6 unaffected pectoralis major muscle samples. Sample 424183 was excluded from differential expression analysis due to an extreme outlier expression pattern.

Sample	Affected/ Unaffected	Total Reads	Paired-end reads after trimming	Mapped reads (Gallus_gallus-5.0)	Mapped reads (GRCg6a)
424132	Unaffected	16,115,674	16,109,441	12,373,514	12,451,896
424170	Unaffected	22,690,579	22,681,762	15,695,351	15,802,383
424183	Unaffected	19,863,892	19,856,809	15,109,341	15,140,824
424198	Unaffected	23,296,111	23,287,439	17,729,792	17,861,743
424379	Unaffected	19,665,248	19,658,088	15,101,602	15,217,990
424439	Unaffected	20,665,929	20,658,137	15,761,365	15,906,603
424207	Affected	19,102,198	19,094,968	12,531,596	12,839,571
424222	Affected	18,665,648	18,658,831	13,960,980	14,003,237
424225	Affected	15,090,867	15,085,500	11,827,301	11,878,037
424239	Affected	24,066,744	24,057,973	17,148,124	17,254,850
424246	Affected	16,289,902	16,283,971	11,993,993	12,131,613
424259	Affected	18,199,967	18,193,176	13,255,515	13,308,199
424266	Affected	17,338,445	17,331,673	13,155,931	13,241,165
485907	Affected	23,577,735	23,568,852	18,765,289	18,898,406
